# Thermoelectric Properties Regulated by Quantum Size Effects in Quasi-One-Dimensional γ-Graphdiyne Nanoribbons

**DOI:** 10.3390/molecules29143312

**Published:** 2024-07-13

**Authors:** Mi Li, Qiaohan Liu, Yi Zou, Jingang Wang, Chuanqiang Fan

**Affiliations:** College of Science, Liaoning Petrochemical University, Fushun 113001, China; 15698943975@163.com (M.L.); lqh13909818502@163.com (Q.L.); happylele1989@126.com (Y.Z.)

**Keywords:** thermoelectricity, one-dimensional material, thermal conductivity, band, GDYNRs

## Abstract

Using density functional theory combined with the first principles calculation method of non-equilibrium Green’s function (NEGF-DFT), we studied the thermoelectric (TE) characteristics of one-dimensional γ-graphdiyne nanoribbons (γ-GDYNRs). The study found that the thermal conductivity of γ-GDYNRs has obvious anisotropy. At the same temperature and geometrical size, the lattice thermal conductivity of zigzag-edged γ-graphdiyne nanoribbons (γ-ZGDYNRs) is much lower than that of armchair-edged γ-graphdiyne nanoribbons (γ-AGDYNRs). We disclose the underlying mechanism for this intrinsic orientation. That is, γ-AGDYNRs have more phonon dispersion over the entire frequency range. Furthermore, the orientation dependence increases when the width of the γ-GDYNRs decreases. These excellent TE properties allow armchair-edged γ-graphdiyne nanoribbons with a planar width of 1.639 nm (γ-Z(2)GDYNRs) to have a higher power factor and lower thermal conductivity, ultimately resulting in a significantly higher TE conversion rate than other γ-GDYNR structures.

## 1. Introduction

Thermoelectric (TE) materials have attracted wide attention because they can directly convert low-quality waste heat into usable electrical energy [1,2,3,4,5,6]. As we all know, the conversion efficiency is determined by $ZT=S^{2}\sigma T/(\kappa_{e}+\kappa_{l})$. Where S, σ, T are the Seebeck coefficient, electrical conductivity, and absolute temperature, respectively. Thermal conductivity (κ) includes electronic ($\kappa_{e}$) and lattice thermal conductivity ($\kappa_{l}$) [7]. In order to maximize the TE property, the material should have low κ, high σ, and a large S. However, these parameters are coupled to each other [8]. Therefore, determining the total controllability of the thermoelectric conversion efficiency is challenging.

Since the emergence of graphene, a large number of γ-graphyne (γ-GY) and γ-graphdiyne (γ-GDY) family members (γ-GFMs), such as γ-graphyne, γ-graphdiyne, and their nanoribbons (γ-GYNRs and γ-GDYNRs), have been synthesized and characterized [9,10,11]. Unlike graphene, these γ-GFMs are semiconductors that could play a key role in the fabrication of next generation nano-electronic devices. In particular, due to quantum confinement effects, these semiconductor materials can reduce the coupling between S and σ, thereby improving the TE property.

Studies have shown that the phonon thermal conductivity of γ-GFMs is significantly lower than that of graphene due to the existence of carbon–carbon triple bonds [12,13,14,15,16,17]. This also shows that the γ-GFMs are the promising “candidate” for high-performance TE materials [18,19,20]. Among them, γ-GDYNRs are a novel one-dimensional carbon allotrope with a unique π-conjugated structure [21,22,23]. They have a perfect hole distribution and the electronic properties of the adjustable $sp^{2}$, sp hybrid planar frame [24,25]. In recent years, Li et al. successfully synthesized γ-GDYNRs with a width of 60 nm through chemical synthesis [11]. Inspired by this, Ouyang et al. found that the *ZT* of γ-GYNRs decreased with an increase in nanoribbon width and showed strong anisotropy [26]. The *ZT* of γ-GDY can reach 0.45, which is one order of magnitude higher than graphene (0.05) [27,28].

Interestingly, the researchers found that the different geometric structures of the boundary atoms have a significant effect on the TE property of γ-GYNRs. At the same width and length, the κ of γ-GYNRs with the armchair edge is 15% higher than those with the zigzag [29]. In addition to the effect of different edge configurations on the κ of γ-GYNRs, the researchers also found that width is an important factor affecting the thermal conductivity of the material. The κ in γ-GYNRs with the zigzag edge presents a “step effect” with the increase in width [30]. In addition, it was found that the κ of γ-GYNRs is also related to external factors (shear strain, tensile strain, etc.). Under unidirectional tensile strain, the κ decreases by 45% and 60%, respectively, while under shear strain, the thermal conductivity decreases by 12% and 16% [31].

In summary, γ-GDYNRs are one of the important raw materials of nanodevices, so it is necessary to study their TE property. In order to ensure the normal operation of the device and extend the life of the device in practical applications, it is meaningful to study the influence of width, edge configuration, and external factors (axial stress, electric field regulation) on the TE property of γ-GDYNRs. This will also provide an important reference for subsequent research on γ-GDYNRs in nanodevices.

## 2. Results and Discussions

**Figure 1:**
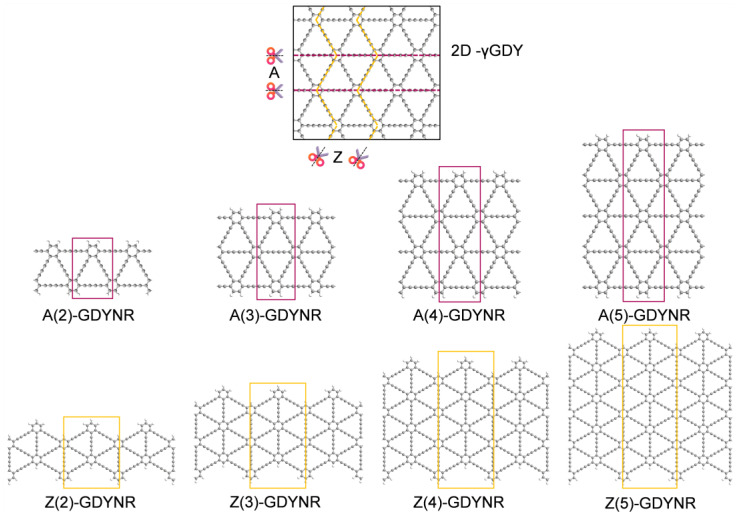
Structural diagrams of γ-A(n)GDYNRs and γ-Z(n)GDYNRs studied in this work. Where *n* represents the index of nanoribbon width, *n* = 2~5.

γ-GDY has a two-dimensional planar structure similar to graphene. This work adjusts its physical property by tailoring in the γ-GDY plane. There are three forms of bonding between carbon and carbon bonds in γ-GDY structure. These are sp−sp, $sp^{2}-sp^{2}$ and $sp-sp^{2}$. Two typical boundary GDYNRs can be obtained by tailoring infinite γ-GDY in different directions; armchair-edged graphdiyne nanoribbons (γ-A(n)GDYNRs) and zigzag-edged graphdiyne nanoribbons (γ-Z(n)GDYNRs). In order to avoid edge reconstruction, the edge carbon atoms were hydrogen passivated after tailoring. The repeat unit cells of γ-A(n)GDYNRs and γ-Z(n)GDYNRs are in the red and yellow wireframes, respectively (Figure 1). γ-A(n)GDYNRs extend in the same direction as the two chains of carbon atoms, at a 30° angle to the other two. In contrast, γ-Z(n)GDYNRs extend at a 30° angle or perpendicular to the direction in which the chain of carbon atoms is arranged.

In this work, quasi-two-dimensional γ-GDY and eight different boundary widths of γ-A(n)GDYNRs and γ-Z(n)GDYNRs were studied (Figure 1). The number of benzene rings *n* represents the index of nanoribbon width. γ-GDYNRs with *n* = 2~5 were selected for calculation, and the optimized cell parameters of γ-A(n)GDYNRs and γ-Z(n)GDYNRs. That is, the widths of repeat cells along the nanoribbon extension direction are 0.946 nm and 1.639 nm, respectively. The optimized cell parameters of quasi-two-dimensional γ-GDY are 0.946 nm. The widths of γ-A(n)GDYNRs (*n* = 2~5) restricted directions were 1.25 nm, 2.07 nm, 2.89 nm, and 3.71 nm, respectively. The widths of the γ-Z(n)GDYNRs’ (*n* = 2~5) restricted direction are 1.92 nm, 2.86 nm, 3.80 nm, 4.74 nm, respectively.

**Figure 2:**
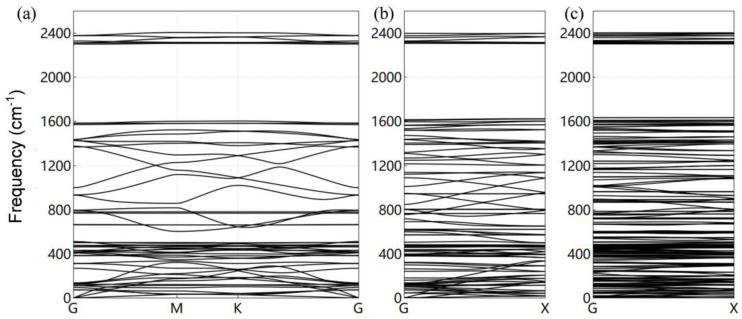
(**a**) Phonon spectrum of γ-GDY; (**b**) Phonon spectrum of γ-A(2)GDYNRs; (**c**) Phonon spectrum of γ-Z(2)GDYNRs.

In Figure 2, the phonon spectra of quasi-two-dimensional γ-GDY, γ-A(n)GDYNRs, and γ-Z(n)GDYNRs are described, respectively. Compared to graphene, two-dimensional γ-GDY has a lower phonon branch (<300 cm^−1^) and introduces some new optical branches at around 2300 cm^−1^. The optical branch between 200~1500 cm^−1^ is quite flat. The phonon spectrum of γ-GDY calculated in this manuscript confirms the observation of two acoustic branches at 1400 cm^−1^ and 2316 cm^−1^.

The comparison in Figure 2b,c show that the phonon band in the γ-A(2)GDYNRs has a larger slope than that in γ-Z(2)GDYNRs. Compared to γ-A(2)GDYNRs, the γ-Z(2)GDYNRs have many flat bands. That is, the phonon dispersion in the γ-A(2)GDYNRs is greater than that in the γ-Z(2)GDYNRs. This difference is mainly due to the unique geometric structure of γ-Z(2)GDYNRs. Compared with γ-A(2)GDYNRs, γ-Z(2)GDYNRs can produce more locally vibrating phonons, because γ-A(2)GDYNRs have more phonon spectral dispersion. At a given frequency, γ-A(2)GDYNRs have more phonon bands than γ-Z(2)GDYNRs. Therefore, it is speculated that γ-A(2)GDYNRs have high lattice thermal conductivity. In addition to the inherent structural properties, boundary conditions are also important factors affecting the thermal anisotropy of γ-GFMs.

**Figure 3:**
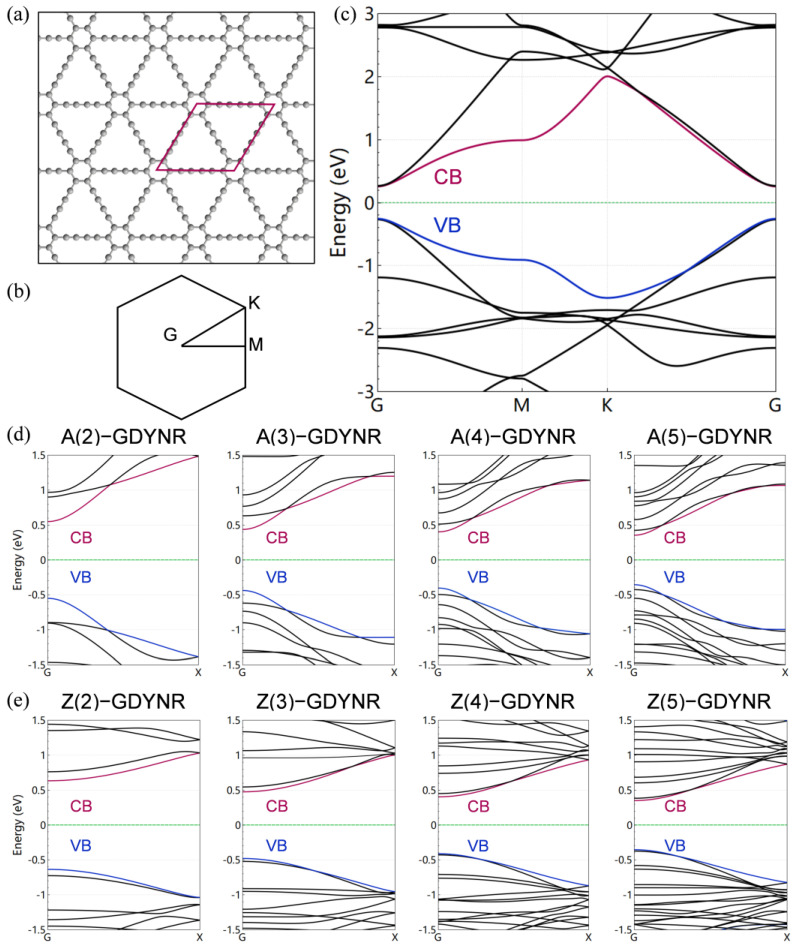
(**a**) Structure diagram of γ-GDY (the red box represents the unit cell of the structure). (**b**) The first Brillouin region of the unit cell in γ-GDY, with high symmetry points (G, M, K, G). (**c**) Electron band structure of γ-GDY (black line, red line (CB), blue line (VB)). (**d**) Electron band structure of γ-A(n)GDYNRs (*n* = 2~5). (**e**) Electron band structure of γ-Z(n)GDYNRs (*n* = 2~5).

Two dimensional γ-GDY has three types of carbon–carbon bonds. The C(sp^2^)–C(sp^2^) bond on the central aromatic ring has a bond length of 1.43 nm. The C(sp^2^)–C(sp) bond connecting C=C and C≡C has a bond length of 0.14 nm. The C(sp)–C(sp) is connected to the C≡C, and the bond length is 0.123 nm. In addition, γ-GDY has the same hexagonal symmetrical structure as graphene [32]. The unique geometric structure determines the electronic structure characteristics of γ-GFMs. Two-dimensional γ-GDY is a direct bandgap semiconductor. The bandgaps are located at the inverted space M(1/2,0,0) and G(0,0,0), respectively (Figure 3b). The bandgap of γ-GDY is 0.52 eV (Figure 3c).

In Figure 3d, e, the bandgaps of the γ-A(n)GDYNRs (*n* = 2~5) are 0.954 eV, 0.817 eV, 0.713 eV, and 0.652 eV, respectively. The bandgaps of the γ-Z(n)GDYNRs (*n* = 2~5) are 1.205 eV, 0.895 eV, 0.703 eV, and 0.652 eV, respectively. It can be observed that the bandwidths of the valence band and conduction band in the γ-Z(n)GDYNRs are obviously larger than those in the γ-A(n)GDYNRs. This also shows that the bonding strength of the atomic orbitals along the nanoribbon is stronger than that along the zigzag nanoribbon. Moreover, the bandgaps of γ-A(n)GDYNRs and γ-Z(n)GDYNRs decrease with increasing nanoribbon width. This variation of the geometric structure has a significant effect on the thermal transport property of γ-GDYNRs.

**Figure 4:**
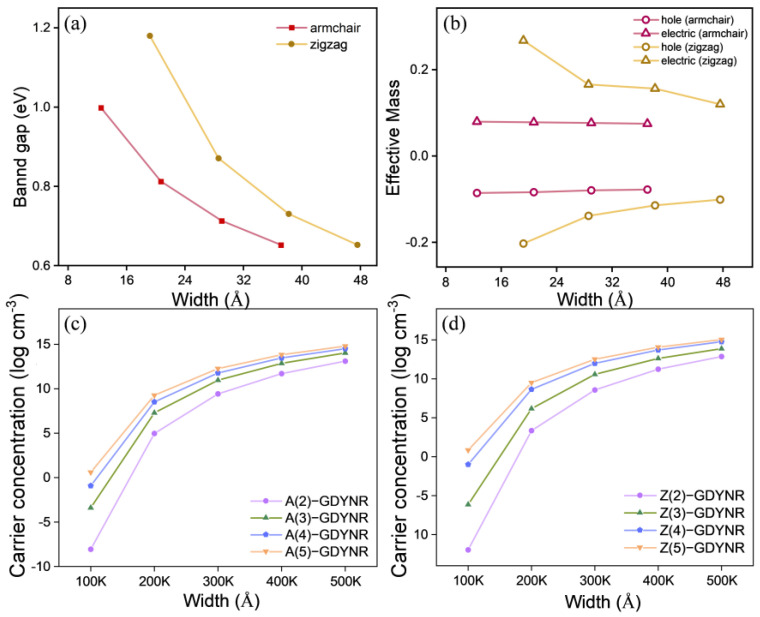
(**a**) The bandgap variations of γ-A(n)GDYNRs and γ-Z(n)GDYNRs (*n* = 2~5). (**b**) The hole (red) and electron (yellow) effective masses of γ-A(n)GDYNRs and γ-Z(n)GDYNRs (*n* = 2~5). (**c**) The carrier concentration with temperature dependence of γ-A(n)GDYNRs (*n* = 2~5). (**d**) The carrier concentration with temperature dependence of γ-Z(n)GDYNRs (*n* = 2~5).

In Figure 4a, the bandgap changes in γ-A(n)GDYNRs and γ-Z(n)GDYNRs at different widths (*n* = 2~5). It can be observed that the bandgaps of γ-A(n)GDYNRs and γ-Z(n)GDYNRs decrease with increasing nanoribbon width. In Figure 4b, the effective masses of γ-A(n)GDYNRs and γ-Z(n)GDYNRs are shown with holes (red) and electrons (yellow) at different widths (*n* = 2~5). It was found that the effective mass of γ-A(n)GDYNRs electrons (hole) is less than that of γ-Z(n)GDYNRs. Figure 4c,d describes the temperature-dependent carrier concentrations of γ-A(n)GDYNRs and γ-Z(n)GDYNRs with different widths (*n* = 2~5). It can be observed that, at low temperatures, thermal excitation is usually limited, making it impossible to generate sufficient carrier concentrations by overcoming the bandgap. As the temperature increases, the carrier concentration is amplified, although the rate of amplification slows down. The carrier concentration increases with increasing nanoribbon width. In addition, the carrier concentration of γ-Z(n)GDYNRs is lower than that of γ-A(n)GDYNRs at the same temperature.

**Figure 5:**
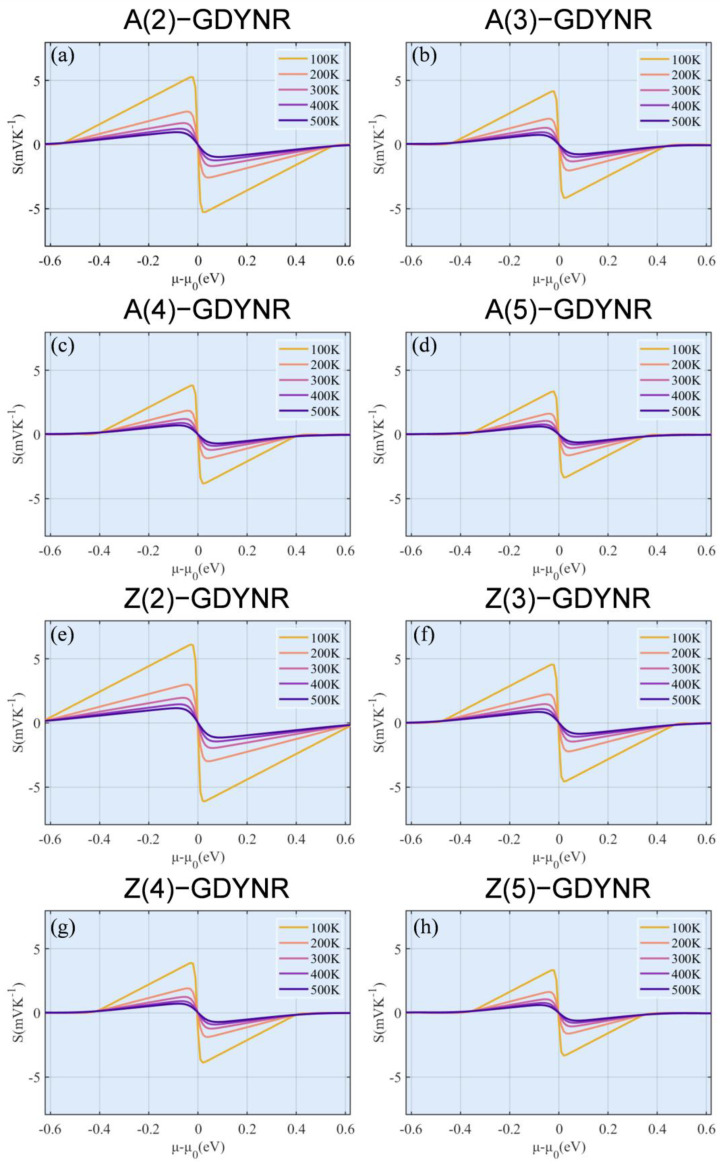
Temperature-dependent Seebeck coefficients as a function of chemical potential for (**a**) γ-A(2)GDYNRs, (**b**) γ-A(3)GDYNRs, (**c**) γ-A(4)GDYNRs, (**d**) γ-A(5)GDYNRs, (**e**) γ-Z(2)GDYNRs, (**f**) γ-Z(3)GDYNRs, (**g**) γ-Z(4)GDYNRs, (**h**) γ-Z(5)GDYNRs.

Subsequently, the transport parameters of γ-GDYNRs with different widths and configurations of the edge were calculated. As can be seen from Figure 5, the Seebeck coefficient shows a clear temperature dependency. Specifically, the Seebeck coefficient decreases with the increase in temperature. In addition, it was found that at the same temperature, the Seebeck coefficients of γ-A(n)GDYNRs and γ-Z(n)GDYNRs show a decreasing trend with increasing width (Figure 5). According to Formula (2), the Seebeck coefficient is not only related to temperature, but also to effective mass and carrier concentration. According to Mott’s relation [33], the Seebeck coefficient is inversely proportional to carrier concentration. Therefore, compared with other γ-GDYNRs with smaller carrier concentrations. The Seebeck coefficients of γ-A(2)GDYNRs and γ-Z(2)GDYNRs are larger (Figure 4c,d). Among them, γ-Z(2)-GDYNRs have the lowest carrier concentration and the largest Seebeck coefficient, which is conducive to realizing the high power factor (PF). 

**Figure 6:**
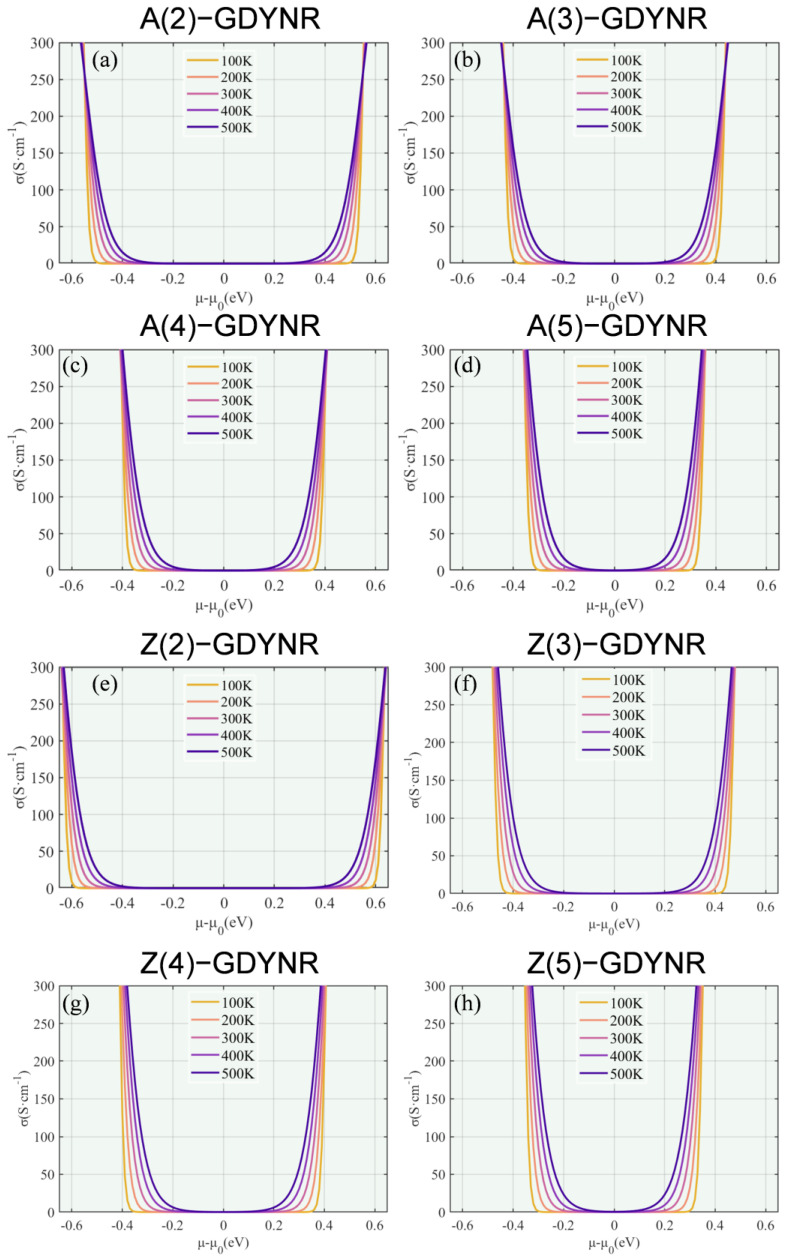
Temperature-dependent electrical conductivity as a function of chemical potential for (**a**) γ-A(2)GDYNRs, (**b**) γ-A(3)GDYNRs, (**c**) γ-A(4)GDYNRs, (**d**) γ-A(5)GDYNRs, (**e**) γ-Z(2)GDYNRs, (**f**) γ-Z(3)GDYNRs, (**g**) γ-Z(4)GDYNRs, (**h**) γ-Z(5)GDYNRs.

Figure 6 shows the chemical function potential of the electrical conductivity of γ-GDYNRs with different boundary configurations and different widths as a function of temperature. Due to the Fermi–Dirac distribution, the electrical conductivity is higher in regions with larger chemical potential. The electrical conductivity of γ-A(n)GDYNRs and γ-Z(n)GDYNRs increases with the increase in width. In addition, γ-GDYNRs have obvious anisotropy. At the same temperature and width, the electrical conductivity of γ-Z(n)GDYNRs is higher than that of γ-A(n)GDYNRs. This phenomenon will further affect the PF.

**Figure 7:**
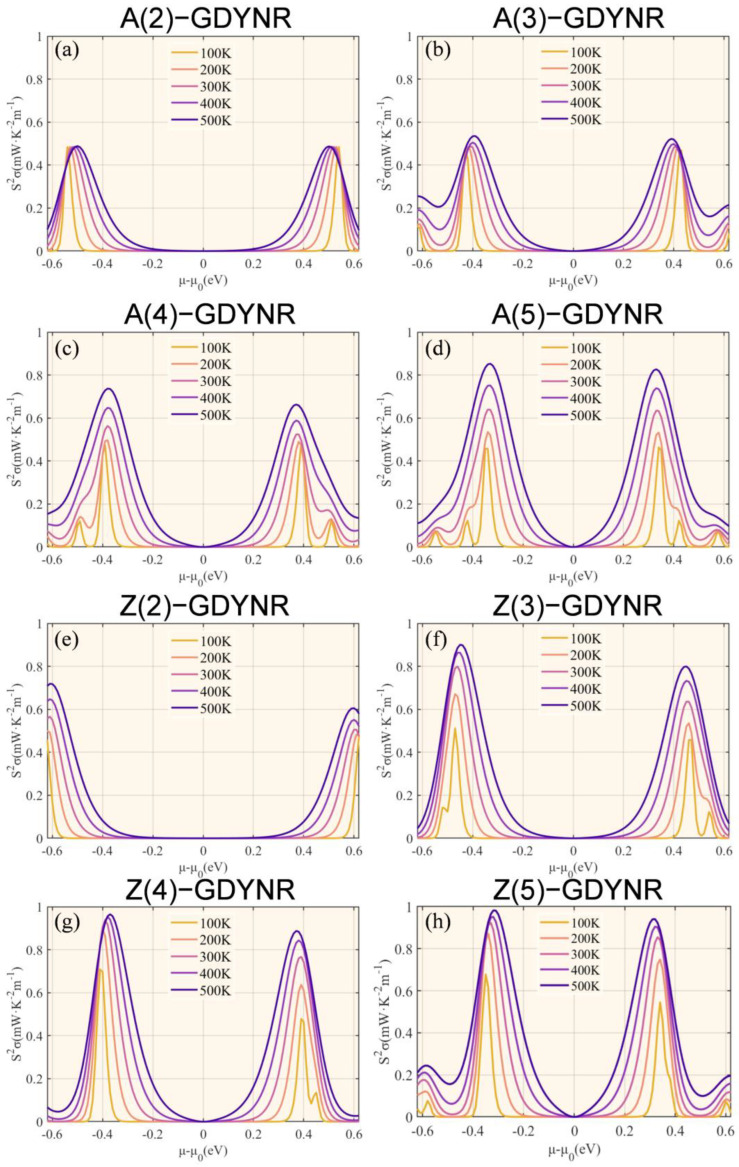
Temperature-dependent power factor (PF) as a function of chemical potential for (**a**) γ-A(2)GDYNRs, (**b**) γ-A(3)GDYNRs, (**c**) γ-A(4)GDYNRs, (**d**) γ-A(5)GDYNRs, (**e**) γ-Z(2)GDYNRs, (**f**) γ-Z(3)GDYNRs, (**g**) γ-Z(4)GDYNRs, (**h**) γ-Z(5)GDYNRs.

The PF of γ-A(n)GDYNRs and γ-Z(n)GDYNRs is calculated from the Seebeck coefficient and electrical conductivity (Figure 7). As shown in Figure 7, the PF shows temperature-dependent behavior. Specifically, the PF increases with the increase in temperature. The greater the PF, the higher the current and voltage generated by the material. Due to the high electrical conductivity of the γ-Z(n)GDYNR structure, compared to other widths of γ-A(n)GDYNRs, the γ-Z(2)GDYNR structure has lower carrier concentration and a higher Seebeck coefficient. Therefore, the γ-Z(n)GDYNRs have a higher PF than the γ-A(n)GDYNRs under the same nanoribbon width.

**Figure 8:**
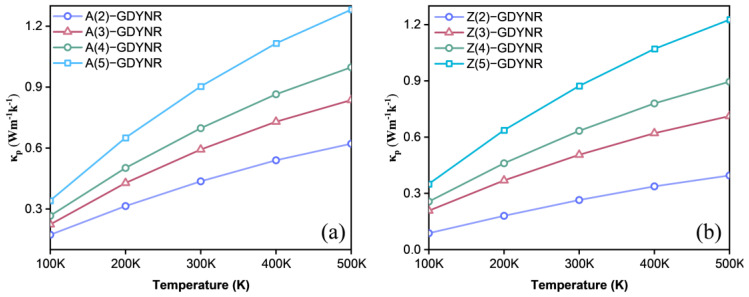
Temperature-dependent lattice thermal conductivity as a function of chemical potential for (**a**) γ-A(n)GDYNRs and (**b**) γ-Z(n)GDYNRs (*n* = 2~5).

Thermal conductivity comprises electronic and lattice thermal conductivity. It can be seen from Figure S6 and Figure 8 that the thermal conductivity of the γ-GDYNRs is mainly contributed by lattice vibration, that is, phonons. In Figure S6, the temperature-dependent chemical potential function of electronic thermal conductivity for γ-A(n)GDYNRs and γ-Z(n)GDYNRs is shown. According to Formula (5), the electronic thermal conductivity is more sensitive to temperature, relative to electrical conductivity. Moreover, the electronic thermal conductivity of γ-GDYNRs showed an increasing trend with the increase in temperature (Figure S6). At 500 K, the electron thermal conductivity increases with the width of γ-A(n)GDYNRs and γ-Z(n)GDYNRs.

For γ-A(2)GDYNRs, γ-A(3)GDYNRs, γ-A(4)GDYNRs, and γ-A(5)GDYNRs at 500 K, the electrical conductivity of thte *p*-type is divided into 0.045 Wm^−1^·K^−1^, 0.075 Wm^−1^·K^−1^, 0.103 Wm^−1^·K^−1^, and 0.130 Wm^−1^·K^−1^ (Figure S6a–d). For γ-Z(2)GDYNRs, γ-Z(3)GDYNRs, γ-Z(4)GDYNRs, and γ-Z(5)GDYNRs at 500 K, the electronic thermal conductivity of the *p*-type is 0.075 Wm^−1^·K^−1^, 0.086 Wm^−1^·K^−1^, 0.093 Wm^−1^·K^−1^, and 0.115 Wm^−1^·K^−1^, respectively (Figure S6e–h). The results show that the electronic thermal conductivity of γ-GDYNRs has strong anisotropy.

Figure 8 shows the lattice thermal conductivity of γ-A(n)GDYNRs and γ-Z(n)GDYNRs at different temperatures. It was found that the lattice thermal conductivity of γ-GDYNRs increases along with the increase in temperature, but as the temperature rises, its growth gradually slows down. At the same temperature, the lattice thermal conductivity increases with the width of γ-A(n)GDYNRs and γ-Z(n)GDYNRs. In γ-A(2)GDYNRs, the lattice thermal conductivity of the *p*-type at 500 K is 0.621 Wm^−1^·K^−1^ (Figure 8a). In γ-Z(2)GDYNRs, the lattice thermal conductivity of the *p*-type at 500 K is 0.395 Wm^−1^·K^−1^ (Figure 8b). It can be seen that the lattice thermal conductivity of γ-Z(2)GDYNRs is much smaller than that of γ-A(2)GDYNRs at the same temperature. The results show that the unique acetylene chain sp carbon atoms of γ-GDYNRs lead to reduced heat transfer efficiency and intense phonon scattering. This is also consistent with the researchers’ finding that the existence of an alkyne bond has a certain inhibitory effect on the lattice thermal conductivity [34].

The conversion efficiency of TE materials can be quantitatively described by the thermoelectric figure of merit (*ZT*) (in Formula (6)). According to the definition of *ZT*, it can be seen that the decrease in thermal conductivity and the increase in PF both play a positive role in promoting the increase in *ZT* value. As shown in Figure 7, Figure 8 and Figure S6, the thermal conductivity of A(n)-GDYNRs and Z(n)-GDYNRs shows a decreasing trend with decreasing width. The power factor shows a decreasing trend with decreasing width. In this case, the influence of thermal conductivity on the *ZT* value is stronger than that of the power factor. It can be inferred that the *ZT* values of A(n)-GDYNRs and Z(n)-GDYNRs show an increasing trend as the width decreases.

**Figure 9:**
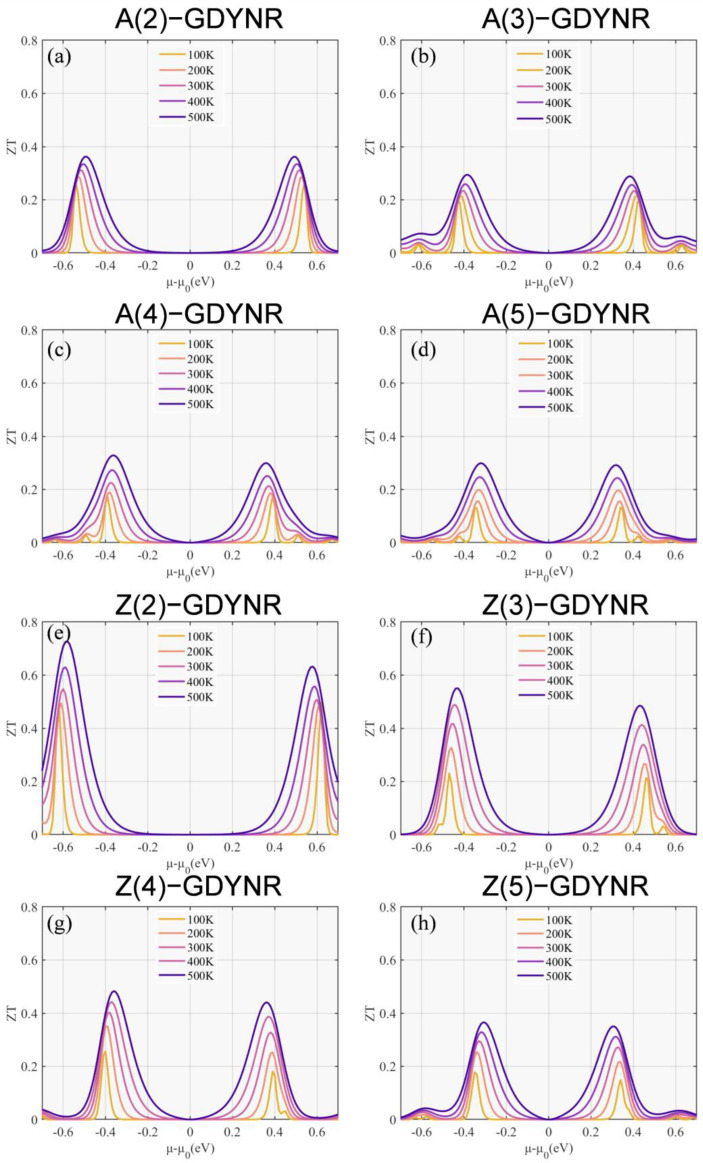
Temperature-dependent *ZT* as a function of chemical potential for (**a**) γ-A(2)GDYNRs, (**b**) γ-A(3)GDYNRs, (**c**) γ-A(4)GDYNRs, (**d**) γ-A(5)GDYNRs, (**e**) γ-Z(2)GDYNRs, (**f**) γ-Z(3)GDYNRs, (**g**) γ-Z(4)GDYNRs, (**h**) γ-Z(5)GDYNRs.

In contrast, as shown in Figure 7, Figure 8 and Figure S6, the thermal conductivity of γ-Z(n)GDYNRs is much lower than of γ-A(n)GDYNRs at the same width. Moreover, the PF of γ-Z(n)GDYNRs is higher than that of γ-A(n)GDYNRs at the same width. It can be inferred that the *ZT* value of γ-Z(n)GDYNRs is higher than that of γ-A(n)GDYNRs at the same width. In other words, in Figure 9, the *ZT* value of carbon-based nanomaterials is influenced by a temperature effect. It can be seen that the *ZT* values of γ-A(n)GDYNRs and γ-Z(n)GDYNRs increase with increasing temperature at different widths. When the temperature remains constant, the *ZT* decreases with the increase in the width of the γ-A(n)GDYNRs and γ-Z(n)GDYNRs. Moreover, for γ-GDYNRs with the same width, the *ZT* value of γ-Z(n)GDYNRs is higher than that of γ-A(n)GDYNRs. This further indicates that the *ZT* value of γ-GDYNRs is highly anisotropic. That is, as the width of the γ-GDYNRs decreases, the orientation dependence becomes stronger, and vice versa. In addition, the larger band gap of the γ-Z(2)GDYNR structure is also conducive to the production of higher *ZT* values (Figure 3e). 

Specifically, the *ZT* value of the *p*-type is 0.363 in the γ-A(2)GDYNRs at 500 K (Figure 9a). The *ZT* value of the *p*-type is 0.328 in the γ-A(3)GDYNRs at 500 K (Figure 9b). The *ZT* value of the *p*-type is 0.299 in the γ-A(4)GDYNRs at 500 K (Figure 9c). The *ZT* value of the *p*-type is 0.294 in the γ-A(5)GDYNRs at 500 K (Figure 9d). The *ZT* value of the *p*-type is 0.727 in the γ-Z(2)GDYNRs at 500 K (Figure 9e). The *ZT* value of the *p*-type is 0.551 in the γ-Z(3)GDYNRs at 500 K (Figure 9f). The *ZT* value of the *p*-type is 0.483 in the γ-Z(4)GDYNRs at 500 K (Figure 9g). The *ZT* value of the *p*-type is 0.366 in the γ-Z(5)GDYNRs at 500 K (Figure 9h).

In addition, external conditions such as applied electric field and axial stress can also be used to regulate the photo-thermoelectric properties of γ-GDYNRs. The results show that when applying electric field or axial stress to γ-A(2)GDYNRs and γ-Z(2)GDYNRs, the TE conversion of γ-A(2)GDYNRs increases first and then decreases. When external electric fields are applied to γ-A(2)GDYNRs and γ-Z(2)GDYNRs, their TE conversion rates peak at 10 V. When the temperature is 500 K, the *ZT* of γ-A(2)GDYNRs is 0.33 (Figure S9b). When the temperature is 500 K, the *ZT* of γ-Z(2)GDYNRs is 0.56 (Figure S9e).

When axial stress is applied to γ-A(2)GDYNRs and γ-Z(2)GDYNRs, the TE conversion rate increases first and then decreases. γ-A(2)GDYNRs has a peak TE conversion rate when applied to +1.5 GPa (*ZT* = 0.61 at 500 K). (Figure S10c) and γ-Z(2)GDYNRs has a peak TE conversion rate when applied to −1 GPa (*ZT* = 1.1 at 500 K) (Figure S10e). Therefore, we conclude that the TE conversion rate is not sensitive to the applied electric field compared to the applied axial stress.

## 3. Method

This work is based on density functional theory combined with the first principles calculation method of non-equilibrium Green’s function (NEGF-DFT). In this work, a theoretical study was carried out to regulate the TE property of one-dimensional γ-GDYNRs by quantum size effect.

Armchair-edged graphdiyne nanoribbons (γ-A(n)GDYNRs) and zigzag-edged graphdiyne nanoribbons (γ-Z(n)GDYNRs) of different widths (*n* = 2~5) were comprehensively optimized [35] using the software package QuantumATK-2019.12 (fermitech, Beijing, China) [36]. The Perdew–Burke–Ernzerhof functional (GGA-PBE), combined with the density functional theory and without damp (DFT-D3) dispersion correction, were used to optimize the structure and ensure that the force on all atoms was less than 0.01 eV Å^−1^. In the calculations, the DFT-LCAO module used 80 Hartree cutoff energies, which comprise a linear combination of pseudo-potential and atomic orbitals [37]. The convergence criterion for the total energy was set at 10^−5^ eV and the K-mesh was 1 × 1 × 3. A vacuum layer of 15 Å was applied in the direction perpendicular to the ribbon plane and between the neighboring ribbons.

Both the electronic and thermal transport properties were evaluated using Nanodcal code [38] with the NEGF-DFT theoretical method. In the calculation, the K-mesh 1 ×1 × 12. When the self-consistent calculation was completed, a scissor correction [39,40] was applied to eliminate the underestimation of the energy gap for the GGA functional. These calculation details were verified to provide accurate results [41].

The carrier concentration was calculated as follows:

(1) $$n=2{\left(\frac{k_{B}T}{2\pi \hbar^{2}}\right)}^{\frac{3}{2}}{\left(m_{h}•m_{e}\right)}^{\frac{3}{4}}\exp\left(-\frac{E_{g}}{2k_{B}T}\right)$$

where ℏ is the reduced Planck’s constant, $m_{h}$ and $m_{e}$ represent the effective mass of holes and electrons, respectively, and $E_{g}$ represents the band gap [33].

The Seebeck coefficient was calculated as follows:

(2) $$S=8\pi^{2}k_{B}^{2}m^{*}T{\left(\pi /3n\right)}^{2/3}/3e\hbar^{2}\propto m^{*}T/n$$

where ℏ is Planck’s constant, $k_{B}$ is Boltzmann’s constant, $m^{*}$ represents the effective mas, and n represents the carrier concentration. The unit of the Seebeck coefficient is mVK^−1^, where V is volt, K is kelvin, and m is meter [42].

The electrical conductivity σ was calculated as follows:

(3) σ=1/ρ

Electrical conductivity is a parameter used to describe the ease of charge flow in a substance. The standard unit of electrical conductivity is Siemens per centimeter (S·cm^−1^) which is the reciprocal of the resistivity (ρ). Electrical conductivity is highly dependent on temperature. The electrical conductivity of metal decreases with an increase in temperature. The electrical conductivity of a semiconductor increases with increasing temperature. Over a range of temperatures, conductivity can be approximated to be proportional to temperature [43].

The lattice thermal conductivity $\kappa_{l}$ is calculated as follows:

(4) $$\kappa_{l}=C_{V}\nu \lambda /3$$

where $C_{V}$ is the lattice heat capacity, which is significantly decreased with the decrease in temperature (T). Where $C_{V}\propto T^{3}$, λ is the mean free path. In a typical solid, $C_{V}$ is 3 $Nk_{B}$, where N is the number of particles and $k_{B}$ is the Boltzmann constant. However, $C_{V}$ in liquids is reduced to 2~2.5 $Nk_{B}$ [44]. The increase in the period length of the superlattice can relatively decrease the mean free path (λ), which can overcome the shortcoming of λ being larger with the decrease in temperature.

The electronic thermal conductivity follows the Wiedemann−Franz law [45] and is calculated as follows: where L is the Lorenz number, $\kappa_{e}$ is directly proportional to σ. The unit of the thermal conductivity is Wm^−1^K^−1^, where W is watt, K is kelvin, and m is meter.

(5) $$\kappa_{e}=L\sigma T$$

The conversion efficiency of TE materials can be quantitatively described by the TE figure of merit. The *ZT* of GDYNRs is calculated using the transmission parameters obtained above. The calculation formula is as follows:

(6) $$ZT=\frac{S^{2}\sigma T}{\left(\kappa_{e}+\kappa_{l}\right)}$$

where S is the Seebeck coefficient, σ is electrical conductivity, T is temperature, $\kappa_{e}$ is electron thermal conductivity, and $\kappa_{l}$ is lattice thermal conductivity. The *ZT* value is a dimensionless parameter, and materials with a large *ZT* value are efficient TE materials.

## 4. Conclusions

To summarize, we have presented the electronic, phonon, and TE properties of γ-GDYNRs by using density functional theory combined with NEGF-DFT. γ-GDYNRs with different widths and edge configurations are direct bandgap semiconductors with unique electronic structures. Moreover, the band gap decreases with the increase in nanoribbon width. This geometric variation has a significant effect on the TE property of γ-GDYNRs. It was found that the increase in the TE property of γ-GDYNRs with the same edge configuration is mainly due to the decrease in $\kappa_{l}$ caused by strong phonon scattering which is caused by the size effect. The effect is much greater than the effect of the PF weakening on the TE property of γ-GDYNRs. In contrast, the TE property of γ-GDYNRs at the same width is improved mainly from the following two aspects. That is, the boundary effect leads to the decrease in $\kappa_{l}$ caused by strong phonon scattering and the enhancement of PF. For γ-GDYNRs with the same width, the *ZT* of γ-Z(n)GDYNRs is higher than that of γ-A(n)GDYNRs. At the same time, with the increase in the size of the geometric structure, the boundary effect weakens and the influence of boundary scattering on the whole weakens. The γ-Z(2)GDYNRs have a high *ZT* which can reach 0.727 at 500 K. The results show that by adjusting the boundary configuration of γ-GDYNRs, the width can improve the TE property of materials. This also provides theoretical support for the TE application of γ-GDYNRs.

## Data Availability

Data are contained within the article and Supplementary Materials.

## Supplementary Materials

The following supporting information can be downloaded at: https://www.mdpi.com/article/10.3390/molecules29143312/s1. Figure S1: Transverse electric fields modulation γ-GDYNRs; Figure S2: Modulating the bandgaps of γ-GDYNRs by transverse electric fields; Figure S3: Modulating the Seebeck coefficients of γ-GDYNRs by transverse electric fields; Figure S4: Modulating the electrical conductivity of γ-GDYNRs by transverse electric fields; Figure S5: Modulating the power factor (PF) of γ-GDYNRs by transverse electric fields; Figure S6: Temperature dependent lattice thermal conductivity as a function of chemical potential for γ-GDYNRs; Figure S7: Modulating the lattice thermal conductivity of γ-GDYNRs by transverse electric fields; Figure S8: Modulating the electronic thermal conductivity of γ-GDYNRs by transverse electric fields; Figure S9: Modulating the *ZT* of γ-GDYNRs by transverse electric fields; Figure S10: Modulating the *ZT* of γ-GDYNRs by axial stress.
