# Supplementary material for: Thermoelectric Properties Regulated by Quantum Size Effects in Quasi-One-Dimensional γ-Graphdiyne Nanoribbons
Source: Molecules. 2024 Jul 13;29(14):3312. doi: 10.3390/molecules29143312 (PMC11279214; doi:10.3390/molecules29143312)
Supplement: Supplementary file 1 [file molecules-29-03312-s001.zip › molecules-3069666-supplementary.pdf]

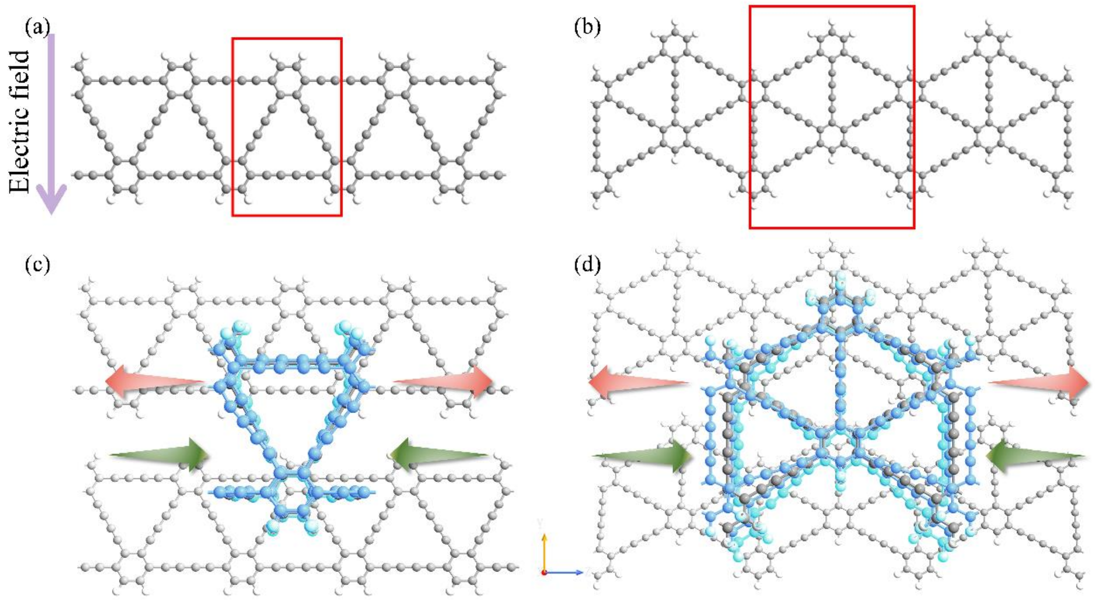

Figure S1. (a) Transverse electric fields modulation γ-A(2)GDYNRs structure diagram; (b) Transverse electric fields modulation γ-Z(2)GDYNRs structure diagram; (c) Axial stress modulation γ-A(2)GDYNRs structure diagram; (d) Axial stress modulation γ-Z(2)GDYNRs structure diagram.

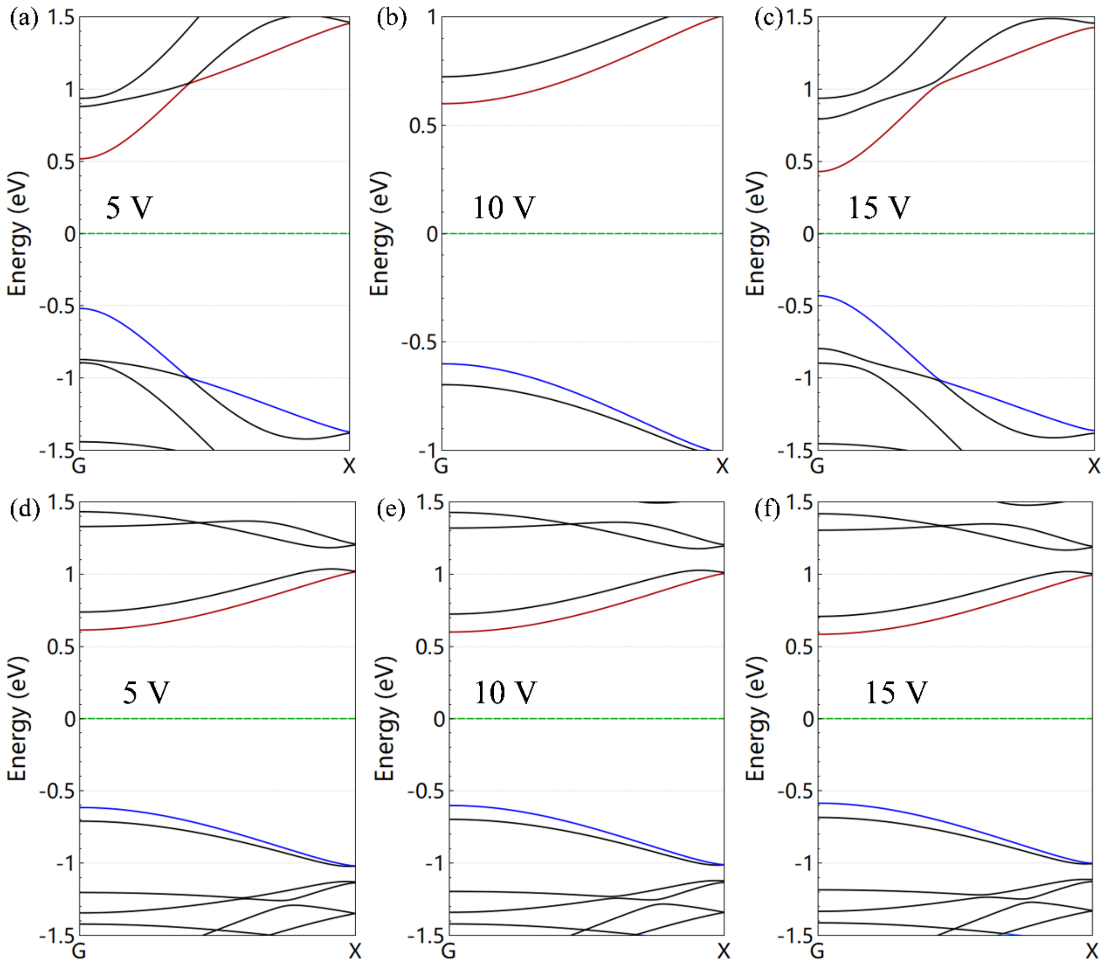

Figure S2. Modulating the bandgaps of γ-A(2)GDYNRs by transverse electric fields. (a) 5 V; (b) 10 V; (c) 15 V; Modulating the bandgaps of γ-Z(2)GDYNRs by transverse electric fields. (d) 5 V; (e) 10 V; (f) 15 V.

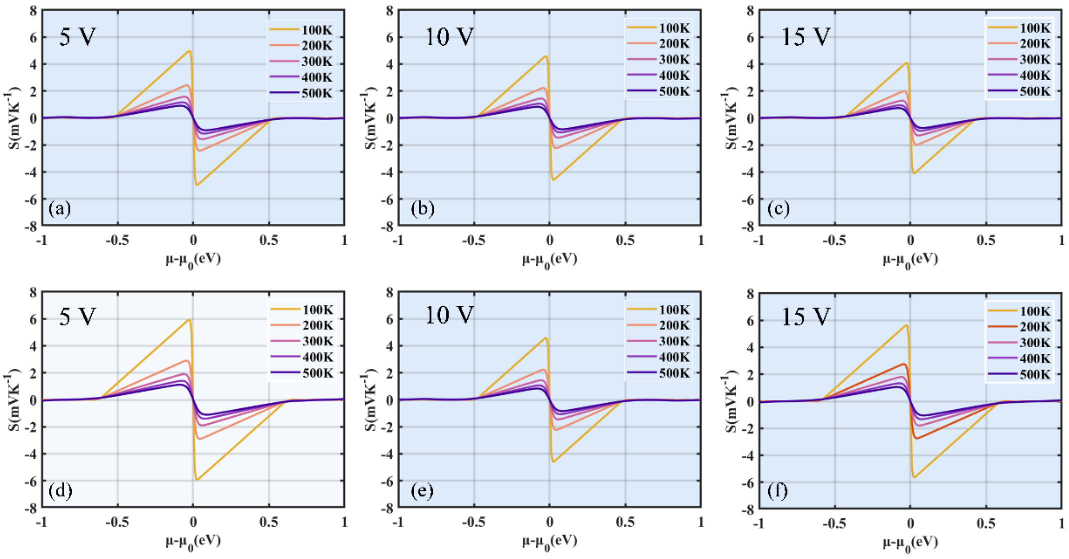

Figure S3. Modulating the Seebeck coefficients of γ-A(2)GDYNRs by transverse electric fields. (a) 5 V; (b) 10 V; (c) 15 V; Modulating the Seebeck coefficients of γ-Z(2)GDYNRs by transverse electric fields. (d) 5 V; (e) 10 V; (f) 15 V.

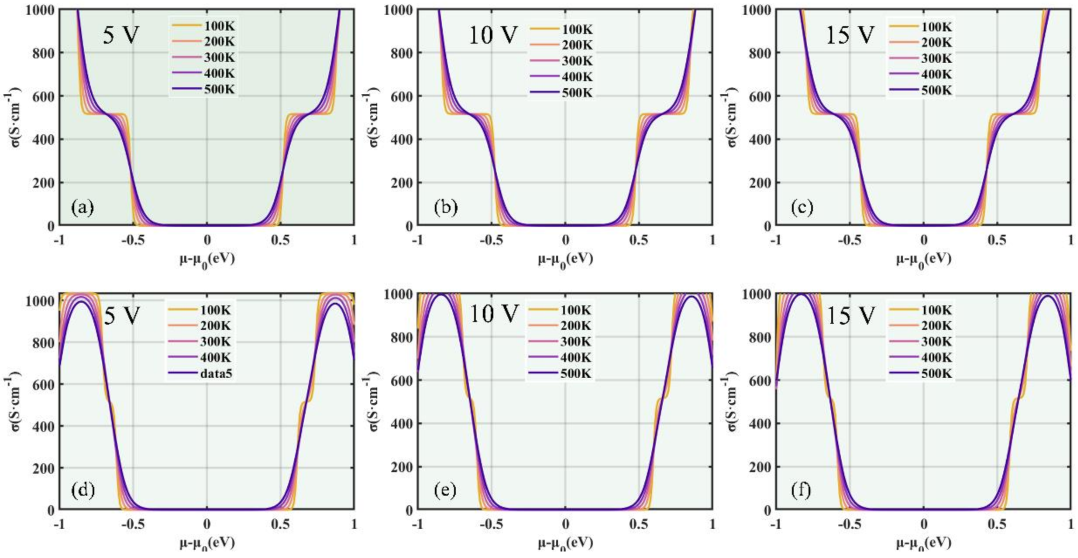

Figure S4. Modulating the electrical conductivity of γ-A(2)GDYNRs by transverse electric fields. (a) 5 V; (b) 10 V; (c) 15 V; Modulating the electrical conductivity of γ-Z(2)GDYNRs by transverse electric fields. (d) 5 V; (e) 10 V; (f) 15 V.

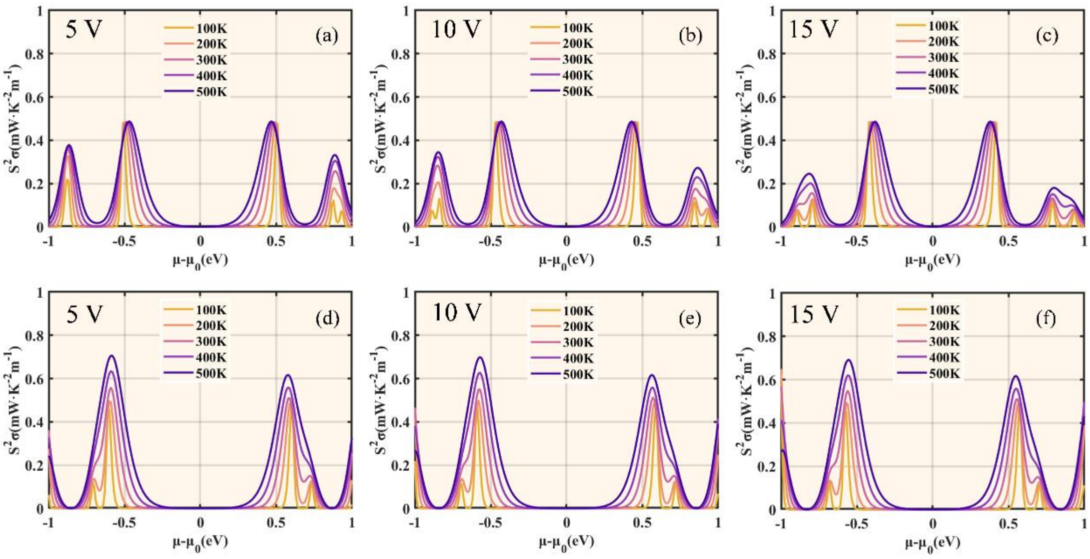

Figure S5. Modulating the power factor (PF) of γ-A(2)GDYNRs by transverse electric fields. (a) 5 V; (b) 10 V; (c) 15 V; Modulating the power factor (PF) of γ-Z(2)GDYNRs by transverse electric fields. (d) 5 V; (e) 10 V; (f) 15 V.

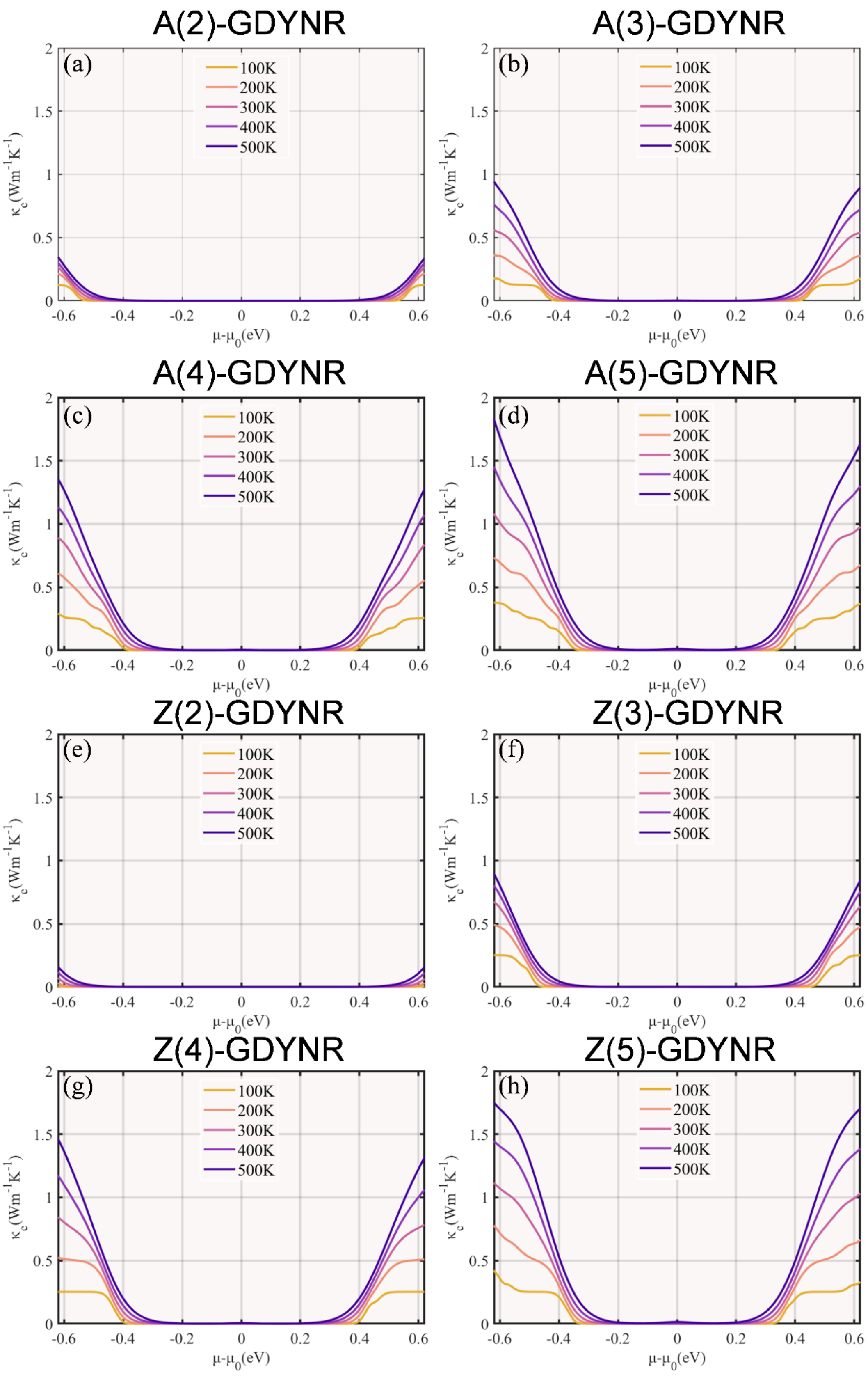

Figure S6. Temperature dependent lattice thermal conductivity as a function of chemical potential for (a) γ-A(2)GDYNRs; (b) γ-A(3)GDYNRs; (c) γ-A(4)GDYNRs; (d) γ-A(5)GDYNRs; (e) γ-Z(2)GDYNRs; (f) γ-Z(3)GDYNRs; (g) γ-Z(4)GDYNRs; (h) γ-

Z(5)GDYNRs.

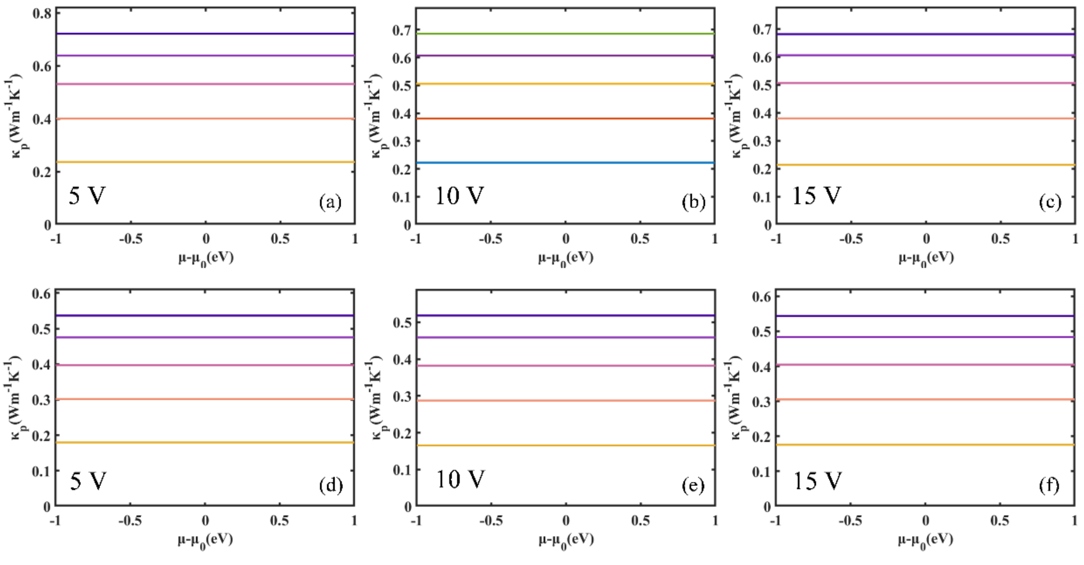

Figure S7. Modulating the lattice thermal conductivity of γ-A(2)GDYNRs by transverse electric fields. (a) 5 V; (b) 10 V; (c) 15 V; Modulating the lattice thermal conductivity of γ-Z(2)GDYNRs by transverse electric fields. (d) 5 V; (e) 10 V; (f) 15 V.

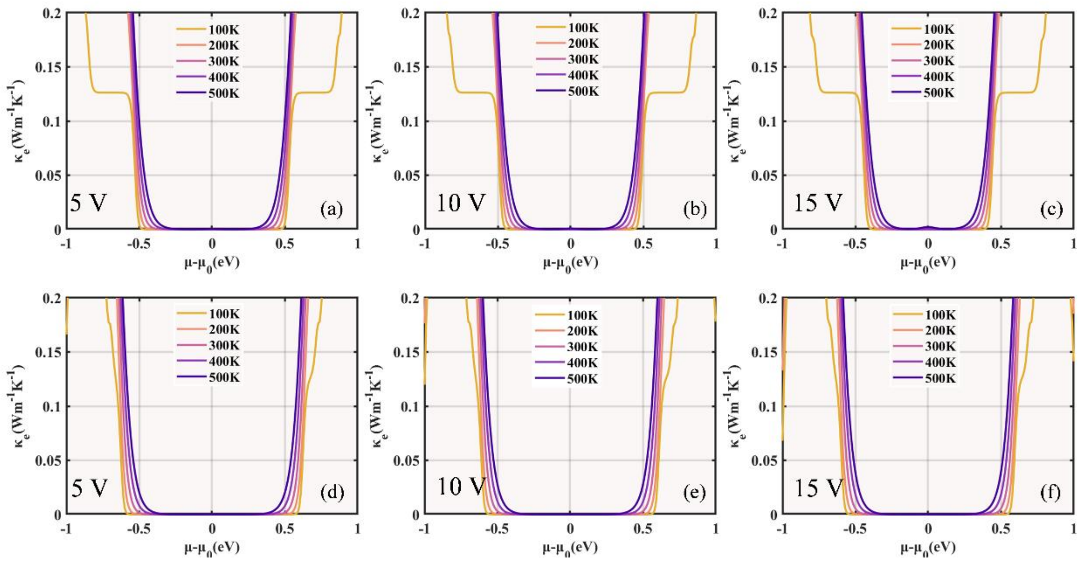

Figure S8. Modulating the electronic thermal conductivity of γ-A(2)GDYNRs by transverse electric fields. (a) 5 V; (b) 10 V; (c) 15 V; Modulating the electronic thermal conductivity of γ-Z(2)GDYNRs by transverse electric fields. (d) 5 V; (e) 10 V; (f) 15 V.

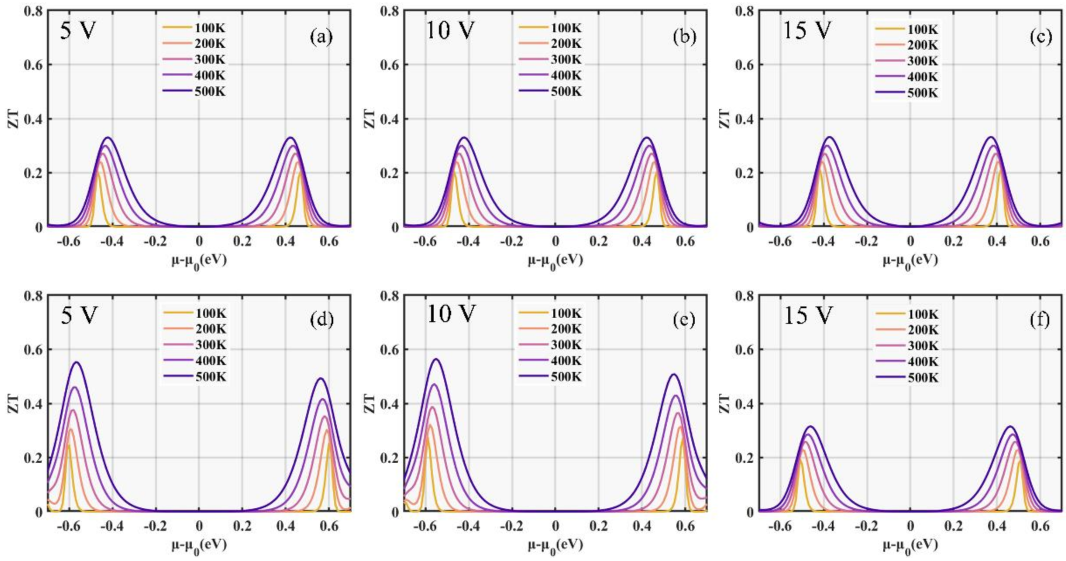

Figure S9. Modulating the *ZT* of γ-A(2)GDYNRs by transverse electric fields. (a) 5 V; (b) 10 V; (c) 15 V; Modulating the *ZT* of γ-Z(2)GDYNRs by transverse electric fields. (d) 5 V; (e) 10 V; (f) 15 V.

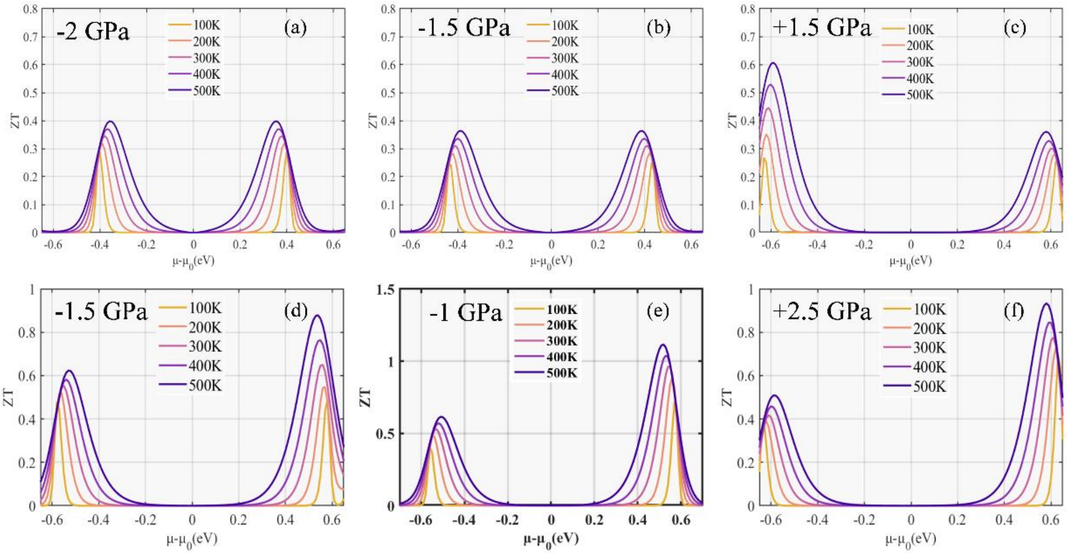

Figure S10. Modulating the *ZT* of γ-A(2)GDYNRs by axial stress. (a) -2 GPa; (b) -1.5 GPa; (c) +1.5 GPa; Modulating the *ZT* of γ-Z(2)GDYNRs by axial stress. (d) -1.5 GPa; (e) -1 GPa; (f) +2.5 GPa.

Convergence test file of γ-A(2)GDYNRs. **convergence.log**
